# Candidates of Genomic Tests in HR+/HER2- Breast Cancer Patients With 1-2 Positive Sentinel Lymph Node Without Axillary Lymph Node Dissection: Analysis From Multicentric Cohorts

**DOI:** 10.3389/fonc.2021.722325

**Published:** 2021-08-05

**Authors:** Zhao Bi, Jia-Jian Chen, Peng-Chen Liu, Peng Chen, Wei-Li Wang, Yan-Bing Liu, Chun-Jian Wang, Peng-Fei Qiu, Qing Lv, Jiong Wu, Yong-Sheng Wang

**Affiliations:** ^1^Shandong Cancer Hospital and Institute, Shandong First Medical University and Shandong Academy of Medical Sciences, Jinan, China; ^2^Shanghai Cancer Center, Fudan University, Shanghai, China; ^3^Department of Breast Surgery, Clinical Research Center for Breast, West China Hospital, Sichuan University, Chengdu, China; ^4^Cheeloo College of Medicine, Shandong University, Jinan, China

**Keywords:** breast cancer, genomic tests, sentinel lymph node biopsy, nomogram, de-escalation

## Abstract

**Background:**

The genomic tests such as the MammaPrint and Oncotype DX test are being gradually applied for hormone receptor positive/HER-2 negative (HR+/HER2-) breast cancer patients with up to three positive axillary lymph nodes (ALNs). The first results from RxPONDER trial suggested that Oncotype DX could be applied to patients with 1-2 positive sentinel lymph nodes (SLNs) without axillary lymph node dissection (ALND), which constituted 37.4% of the intent-to-treat population. However, there was no distinctive research on how to apply genomic tests precisely to HR+/HER2- patients with 1-2 positive SLNs without ALND. The purpose was to construct a nomogram using the multi-center retrospective data to predict precisely which HR+/HER2- candidates with 1-2 positive SLNs could be subjected to genomic tests (≤ 3 positive lymph nodes).

**Methods:**

We conducted a retrospective analysis of 18,600 patients with stage I-III breast cancer patients treated with sentinel lymph node biopsy (SLNB) in Shandong Cancer Hospital, Fudan University Shanghai Cancer Center, and West China Hospital. The univariate and multivariate logistic regression analysis was conducted to identify the independent predictive factors of having ≤ 3 positive nodes among patients with 1-2 positive SLNs. A nomogram was developed based on variables in the final model with *p*<0.05. Calibration of the nomogram was carried out by internal validation using the bootstrap resampling approach and was displayed using a calibration curve. The discrimination of the model was evaluated using the ROC curve.

**Results:**

Based on the database of the three institutions, a total of 18,600 breast cancer patients were identified undergoing SLNB between May 2010 and 2020. Among the 1817 HR+/HER2- patients with 1-2 positive SLNs undergoing ALND, 84.2% harbored ≤ 3 totals metastatic ALNs. The multivariate logistic regression analysis identified imaging abnormal nodes (OR=0.197, 95%CI: 0.082-0.472), the number of positive SLNs (OR=0.351, 95%CI: 0.266-0.464), the number of negative SLNs (OR=1.639, 95%CI: 1.465-1.833), pathological tumor stage (OR=0.730, 95%CI: 0.552-0.964), and lympho-vascular invasion (OR=0.287, 95%CI: 0.222-0.398) as independent predictors for the proportion of patients with ≤ 3 total metastatic ALNs (all *p*<0.05). These five predictors were used to create a predictive nomogram. The AUC value was 0.804 (95%CI: 0.681-0.812, *p*<0.001). The calibration curve showed a satisfactory fit between the predictive and actual observation based on internal validation with a bootstrap resampling frequency of 1000.

**Conclusion:**

The nomogram based on the multi-centric database showed a good accuracy and could assist the oncologist in determining precisely which HR+/HER2- candidates with 1-2 positive SLNs without ALND could perform genomic tests. In the era of SLNB and precision medicine, the combined application of genomic tests and SLNB could provide patients with a better strategy of dual de-escalation management, including the de-escalation of both surgery and systemic treatment.

## Background

The status of axillary lymph nodes (ALNs) involvement in patients with early breast cancer is among the most essential prognostic factors, while playing a significant role in the decision making for adjuvant systemic therapy (1). Historically, axillary lymph node dissection (ALND) remains the standard of management for patients with metastatic sentinel lymph nodes (SLNs) (2). However, this situation was changed with the publication of several randomized, controlled trials such as ACSOG Z0011, AMAROS and OTOASOR. No difference in axillary regional recurrence (RR) and overall survival (OS) were detected with or without ALND for early breast cancer patients with limited SLN involvement (1-2 positive SLNs) (3–5). But this change in the concept of axillary management prevents us from fully assessing the status of ALN metastases.

The genomic tests such as the 70-gene expression assays (MammaPrint) and 21-gene expression assays (Oncotype DX) may be used to inform decisions on withholding adjuvant chemotherapy in HR+/HER2- breast cancer patients with high clinical risk due to their ability to identify populations with a good prognosis and potentially limited chemotherapy benefit (6). The MINDACT trial enrolled 80.7% of hormone receptor positive/HER-2 negative (HR+/HER2-) patients, while in RxPONDER trial, all enrolled population were HR+/HER2- patients. At the same time, the MammaPrint assays had be gradually applied for patients with up to three positive ALNs. The first results from RxPONDER trial showed that postmenopausal women with 1-3 positive nodes and recurrence score (RS) 0-25 can likely safely forego adjuvant chemotherapy without compromising invasive disease-free survival (iDFS) (7). However, full assessment of ALN metastasis status is a prerequisite for application of the MammaPrint and Oncotype DX. As the omission of ALND has been widely applied for patients with 1-2 positive SLNs, we could not assess the overall ALNs metastases status of these patients through ALND. However, these patients could also have chance to be subjected to MammaPrint and Oncotype DX, and patients with low genomic risk might safely avoid chemotherapy. While in the RxPONDER trial, only 37.4% of HR+/HER2- patients with 1-2 positive sentinel nodes were not required to undergo full ALND (7). So, there was no distinctive research on how to apply genomic tests precisely to HR+/HER2- patients with 1-2 positive SLNs without ALND.

The initial SLNB plus ALND revealed that 5.7% to 18.9% of patients with 1-2 positive SLNs harbored more than three metastatic ALNs, while more than 80% of those patients had ≤ 3 metastatic ALNs (**Table 1**) (2–5, 8, 9). Based on these results, we proposed that the genomic tests are also applicable to most patients with 1-2 positive SLNs who may avoid ALND (10). And those patients at high clinical/low genomic risk might also safely avoid chemotherapy. Therefore, we need to identify precisely which HR+/HER2- patients with 1-2 positive SLNs meet the requirements for genomic tests (≤ 3 positive lymph nodes). Considering of limited nodal information, adjuvant decision making of HR+/HER2- patients need to know both the presence of lymph node involvement and the number of positive nodes. The purpose of this study was to construct a nomogram using the multi-center retrospective data to predict which candidates with 1-2 positive SLNs could be subjected to genomic tests (≤ 3 positive lymph nodes).

**Table 1:** The probability of >3 positive ALNs among different trials.

Study	Daivd et al.	Kim et al.	AMAROS	Z0011	OTOASOR	Our
Breast management	BCS+M	BCS+M	BCS+M	BCS	BCS+M	BCS+M
Axillary management	SLNB→ALND	SLNB+RT *vs.* SLNB→ALND	SLNB+RT *vs.* SLNB→ALND	SLNB only *vs.* SLNB→ALND	SLNB+RT *vs.* SLNB→ALND	SLNB→ALND
Number of ALND	405	1437	300	420	244	3196
>3 ALN+	25.7%	NA	13.0%	13.7%	22.0%	25.0%
>3 ALN+ in 1-2 SLN+ patients	18.9%	5.7%	13.0%	13.7%	NA	15.8%

NA, Not available.

## Patients and Methods

### Patient Characteristics

The medical records of breast cancer patients who underwent surgery in Shandong Cancer Hospital, Fudan University Shanghai Cancer Center, and West China Hospital of Sichuan University between May 2010 and 2020 were retrospectively reviewed. Adult women were included in this study if they 1) had histologically confirmed invasive breast carcinoma; 2) were clinically node-negative (cN0) or cN0 with image positive node (iN+) detected by ultrasound, Morphologic characteristics predictive of imaging positive lymph nodes detected by ultrasound are cortical thickness greater than 2.5-3.0 mm, focal cortical lobulation, loss of the fatty hilum, a round shape, and abnormal cortical blood flow; 3) had undergone lumpectomy or mastectomy plus SLNB and ALND; and 4) harbored pT1-2 and 1-2 metastatic SLNs detected by frozen section, touch preparation, or hematoxylin-eosin staining on permanent section. Patients were not eligible if they had T3-4 primary tumor, bilateral breast cancer, a medical history of previous malignancy, undergone neoadjuvant systemic treatment for the primary breast cancer, or treatment of the axilla by surgery or radiotherapy.

The following clinicopathological data were collected: age; multicenter/multifocality; type of surgery; tumor histopathological type and grade; tumor size and pathological tumor stage; imaging abnormal nodes (cN0/iN+); lympho-vascular invasion (LVI); number of positive and negative SLNs; total number of positive lymph nodes; status of estrogen receptor, progesterone receptor, HER-2 and Ki-67 index. Data on laboratory indices and location of primary tumor were unavailable for the majority of patients in this cohort due to the retrospective study.

This multicenter, retrospective study was registered with the Shandong Cancer Hospital Ethics Committee (No. SDTHEC20110324) and approved by the institutional review boards of participating centers. Written informed consent was obtained from all patients before participation in the study. The study protocol was approved by independent ethics committees at every participating center, and the study was undertaken in full accordance with the Declaration of Helsinki.

### Surgery

Each center participating in the study fulfilled the surgical quality control criteria prior to the study and used the same method to find SLN. The SLNB procedure had to be done with echnetium-99m colloid (^99m^Tc) colloid, preferably combined with blue dye. The ^99m^Tc colloid was injected subcutaneously 3-18 h before the operation with an injection dose of 1.0 mCi (0.5 mCi/mL). After anesthesia was administered, blue dye (2.0 mL for breast-conservation surgery and 4.0 mL for mastectomy) was injected subcutaneously around the tumor 15 minutes before surgery. Fifteen minutes later, the SLNB procedure began. We dissected the axilla along the blue lymphatic vessels, and the lymph nodes marked with blue dye are SLNs. The gamma detector (Neoprobe Corporation) was switched to 27keV, and was used to detect the radioactive hotspot. Lymph nodes including radioactive or blue-stained lymph nodes were excised as SLNs for histopathological evaluation.

The ALND should include inferior to the axillary vein from the latissimus doris muscle laterally to the medial border of the pectoralis minor muscle (level I/II), the level III dissection should be performed in cases with gross disease in level II/III nodes (3). In these three centers, we performed ALND with at least ten nodes from anatomical levels I-III. Local treatment of the breast included breast-conserving surgery and mastectomy.

### Pathological Evaluation

The axillary node evaluation was performed per the standard of care at each institution. Each SLN was examined at multiple histologic levels. After SLN was taken out, the SLN was dissected from adipose tissue and cut into 2-4 blocks. Then these nodes were separately embedded and frozen within optimal cutting tissue media and cut on a standard (-20°C) cryostat, creating 6-8 μm -thick sections, with a minimum of two levels per block. Frozen section analysis was performed after hematoxylin and eosin (H&E) staining of a portion of the frozen nodal tissue. The remaining tissue was fixed in formalin, embedded in paraffin, and stained with H&E for further evaluation.

Tumor deposits were categorized as isolated tumor cells (<0.2mm), micro-metastases (0.2 to 2mm), or macro-metastases (>2mm). Isolated tumor cells, macro-metastases and micro-metastases were all considered as positive lymph nodes.

Positive HR status was defined as at least one percent of tumor cells expressing the receptor. HER-2 status was determined based on the American Society of Clinical Oncology/College of American Pathologists guidelines. Positive HER-2 was defined as HER-2 over-expression (3+) detected by immune-histochemical staining or fluorescence *in situ* hybridization (9). To accurately evaluate the effect of molecular subtypes, patients were further classified into HR+/HER2-, triple negative and HER-2 positive subtype.

### Adjuvant Systemic Therapy

There were no standard indications for offering systemic therapy in the protocol. The actual chemotherapies and endocrine therapies were given according to the newest guidelines. To obtain an objective criterion for the administration of adjuvant therapy, we used the clinicopathologic risk as predicted by Adjuvant! online system and a predefined cut off value of clinical high-and low-risk patients (6).

### Statistical analysis

The continuous variable was divided into two groups according to the optimal cut-off values determined by maximizing the Youden index (sensitivity + specificity - 1) using receiver operating characteristic (ROC) curve analyses. The remaining clinicopathological factors were analyzed as categorical variables. The association of different clinicopathological variables with final lymph node status (≤ 3 positive nodes) was analyzed. Pearson chi-square test or Fisher exact test was used to perform univariate analysis on categorical variables. Multivariable logistic regression analysis was conducted to identify the independent predictive factors of having ≤ 3 positive nodes by using backward stepwise analysis.

The nomogram was developed based on variables in the final model with *p* < 0.05 using “rms” package for R. Calibration of the nomogram was carried out by internal validation using the bootstrap resampling approach and was displayed using a calibration curve. The discrimination of the model was evaluated using the area under the curve (AUC) value of the ROC curve. Statistical analyses were performed using SPSS Statistics 22.0 software (IBM Corporation, Armonk, NY, USA) and R version 3.3.3 software (The R Foundation for Statistical Computing, Austria, Vienna). A *p* < 0.05 was considered statistically significant.

## Results

**Figure 1:**
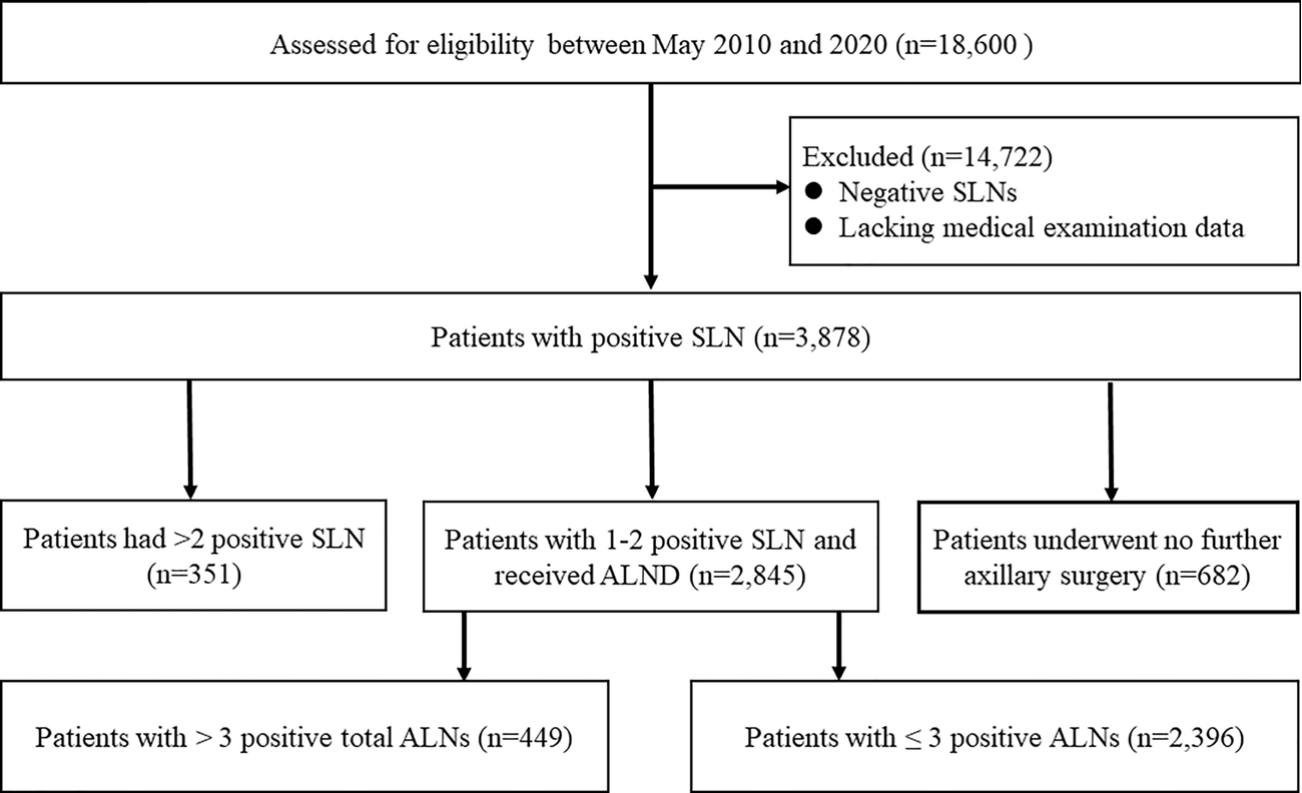
The consort diagram of the trial.

The consort diagram of the study was illustrated in **Figure 1**. Based on the database of the three institutions, we identified a total of 18,600 breast cancer patients who underwent SLNB between May 2010 and 2020. After excluding cases having negative SLNs or lacking medical examination data, we included 3878 women with positive SLNs. Of the initial 3878 patients, 351 were ineligible because of harboring more than two positive SLNs. Among the remaining 3527 patients with 1-2 positive SLNs, 19.3% (682/3527) received no further axillary surgery. Among the remaining 2845 patients, there were 1817 HR+/HER2- patients. Thus, a total of 1817 patients with 1-2 positive SLNs undergoing ALND were included in the final analysis.

The basic characteristics of the patients, tumors, and treatments were listed in **Table 2**. The median age of the patients was 48 years (range 21-80 years). More than half of patients (50.6%) have a pT2 tumor. Notably, among these population, 84.2%, 11.8%, and 4.0% had nodal involvement in one to three nodes (pN1), four to nine nodes (pN2), and more than nine nodes (pN3), respectively.

**Table 2:** Relation between positive SLNs and positive non-SLNs among HR+/HER2- patients.

Pathological positive SLNs	Pathological positive non-SLNs				Total
	0	1	2	≥3	
1	876 (48.2%)	183 (10.0%)	70 (3.9%)	124 (6.8%)	1253
2	296 (16.3%)	105 (5.8%)	52 (2.9%)	111 (6.1%)	564
Total	1172	288	122	235	1817

The number of positive pathological SLNs and positive pathological non-SLNs were summarized in **Table 3**. Out of the 1817 HR+/HER2- patients with 1-2 positive SLNs, 15.8% (287) had more than 3 metastatic ALNs, while the remaining 84.2% had ≤ 3 positive ALNs.

**Table 3:** The clinical characteristics of HR+/HER2- patients.

Characteristics	Patients
Age, median (range), years	49 (21-80)
Tumor size, median(range), cm	2.0 (0.2-5.0)
Pathological Tumor stage	
pT1	945 (52.0%)
pT2	872 (48.0%)
Axillary lymph node metastasis	
1-3	1530 (84.2%)
4-9	214 (11.8%)
>9	73 (4.0%)
Positive SLN	
1	1253 (69.0%)
2	564 (31.0%)
Negative SLN	
0	232 (12.8%)
1	371 (20.4%)
2	492 (27.1%)
3	381 (21.0%)
4	200 (11.0%)
>4	141 (7.8%)
Imaging abnormal nodes	
cN0	1792 (98.6%)
iN1	25 (1.4%)
Tumor type	
Ductal, I	35 (2.0%)
Ductal, II	1216 (66.9%)
Ductal, III	435 (23.9%)
Lobular	74 (4.1%)
Special	57 (3.1%)
Estrogen receptor status	
Positive	1784 (98.2%)
Negative	33 (1.8%)
Progesterone receptor status	
Positive	1636 (90.0%)
Negative	181 (10.0%)
Lymph-vascular invasion	
Yes	828 (45.6%)
No	989 (54.4%)
Type of breast surgery	
Lumpectomy	422 (23.2%)
Mastectomy	1395 (76.8%)
Multifocal/multicenter	
Yes	142 (7.8%)
No	1675 (92.2%)

**Table 4** showed the univariate and multivariate logistic regression analysis of variables associated with ≤ 3 positive nodes. Variables with *p*-value <0.05 in the univariate analysis were assessed for multivariate analysis. The independent predictors of ≤3 positive nodes were comprised of imaging abnormal nodes (OR=0.197, 95%CI: 0.082-0.472, *p*<0.001), the number of positive SLNs (OR=0.351, 95%CI: 0.266-0.464, *p*<0.001), the number of negative SLNs (OR=1.639, 95%CI: 1.465-1.833, *p*<0.001), pathological T stage (OR=0.730, 95%CI: 0.552-0.964, *p*=0.027), and LVI (OR=0.287, 95%CI: 0.222-0.398, *p*<0.001).

**Table 4:** Clinicopathologic characteristics and association with positive total ALNs among HR+/HER2- patients.

Characteristic	1-3 positive ALNs	More than 3 positive ALNs	Univariable Analysis	Multivariable Analysis
			p value	p value
Pathological Tumor stage			<0.001	0.025
pT1	827	118		
pT2	703	169		
Imaging abnormal nodes			<0.001	<0.001
cN0	1516	276		
iN1	14	11		
Positive SLNs			<0.001	<0.001
1	1129	124		
2	401	163		
Negative SLNs			<0.001	<0.001
0	145	87		
1	300	71		
2	420	72		
3	342	39		
4	189	11		
>4	134	7		
Tumor type			0.076	
Ductal, I	31	4		
Ductal, II	1036	180		
Ductal, III	353	82		
Lobular	58	16		
Special	52	5		
Lymph-vascular invasion			<0.001	<0.001
No	195	92		
Yes	633	897		
Estrogen receptor			0.634	
Positive	1503	281		
Negative	27	6		
Progesterone receptor			0.335	
Positive	1382	254		
Negative	148	33		
Multifocal/multicenter			0.719	
Yes	118	24		
No	1412	263		

**Figure 2:**
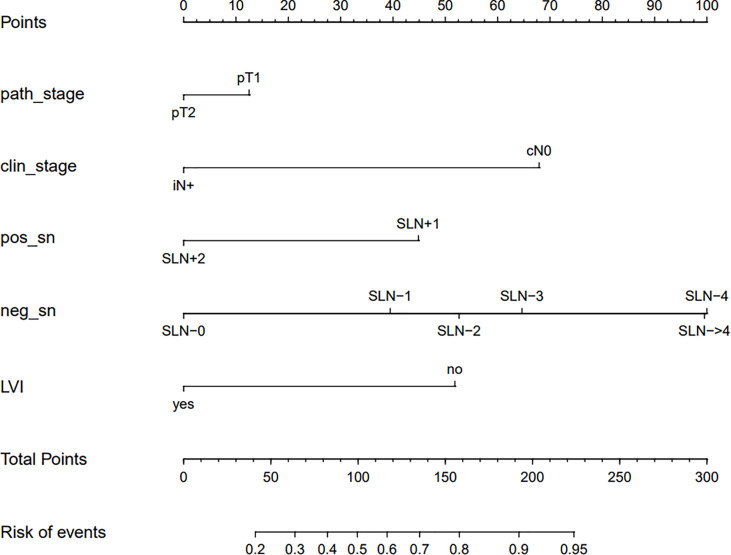
The nomogram to predict patients with ≤ 3 positive total ALNs in HR+/HER2- population with 1-2 positive SLNs. To calculate the probability of ≤ 3 positive ALNs, the scores for the five factors were summed up. And the total scores and bottom risk scale were referenced.

**Figure 3:**
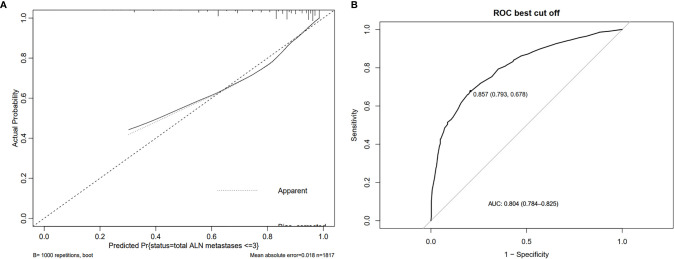
The overall performance and discriminative performance of the nomogram were assessed by the calibration curve and ROC curve analysis, respectively. **(A)** The calibration curve showed a satisfactory fit between the predictive and actual observation. **(B)** The ROC curve of the nomogram.

Based on data obtained from the multivariate analysis, a nomogram was created to predict patients with ≤ 3 positive ALNs in those HR+/HER2- patients with 1-2 positive SLNs (**Figure 2**). To calculate the probability of ≤ 3 positive ALNs, the scores for the five factors were summed up. And the total scores and bottom risk scale were referenced. The overall performance and discriminative performance of the model were assessed by the calibration curve and ROC curve analysis, respectively. Based on internal validation with a bootstrap resampling frequency of 1000, the calibration curve showed a satisfactory fit between the predictive and actual observation (**Figure 3A**). The ROC curve of the nomogram was depicted in **Figure 3B**. The AUC value was 0.804 (95%CI: 0.681-0.812, *p*< 0.001), indicating that the nomogram had a good discriminatory capability.

Meanwhile, the AUC values for prediction of ≤ 3 positive total ALNs were 0.426, 0.598, 0.633, 0.574, and 0.626 for the number of positive SLNs, the number of negative SLNs, LVI, pathological T stage, and imaging abnormal nodes, respectively. These data indicated that the multi-factors model could improve single-factor predictive power.

On the basis of the predicted probability of ≤ 3 positive total ALNs, we calculated the prediction accuracy of different cutoff points. **Table 5** presents the sensitivity, specificity, positive predictive value, negative predictive value, and accuracy of this nomogram at different cutoff points for the entire cohort.

**Table 5:** Classification accuracy for prediction probability at different risk cutoff points for the nomogram.

Predicted probability	Sensitivity (%)	Specificity (%)	PPV (%)	NPV (%)
≥20%	100%	0%	84.2%	0%
≥50%	96.3%	16.4%	86.0%	45.6%
≥75%	84.8%	52.3%	90.4%	39.2%
≥80%	79.9%	64.1%	92.2%	37.4%
≥85%	69.3%	74.9%	93.6%	31.4%

PPV, Positive predictive value; NPV, Negative predictive value.

## Discussion

This study presented a simple nomogram that could be used to predict precisely which HR+/HER2- patients with 1-2 positive SLNs would have ≤3 positive ALNs. The model was developed based on the principles of transparent reporting of a multivariable prediction model for individual prognosis or diagnosis (TRIPOD statement) (11), and it indicated that HR+/HER2- patients with 1-2 positive SLNs had less imaging abnormal nodes, a smaller number of positive SLNs, a greater number of positive SLNs, lower pathological tumor size, and less LVI were more likely to have ≤3 positive ALNs. With an AUC of 0.804 (95%CI: 0.681-0.812), and *via* internal validation using the bootstrap resampling method, the model exhibited sufficient ability to predict ≤ 3 positive ALNs among HR+/HER2- patients with 1-2 positive SLNs. The diversity of our population across many centers adds to the generalizability of this findings.

In the present study, the main predictors included overall pathological size, SLNs tumor burden (characterized by the number of positive SLNs and negative SLNs), imaging abnormal nodes, and LVI. In previous studies, the main predictors for the total number of ALNs metastases ≤ 3 include the primary tumor size, the SLNs tumor burden and LVI (8, 9, 12–16). Most of the patients in these studies had T1-2 stage tumors and had 1-2 positive SLNs. Studies by Katz et al. (13) and Yang et al. (11) found that the location of the primary tumor is also related to the number of ALNs metastases ≤3. However, in our study, due to the retrospective design, the location of the primary tumor is difficult to obtain. As for the variable of imaging abnormal nodes, we also found it was associated with the number of ALNs metastases ≤3. A meta-analysis comprising 4271 patients assessed the proportion of patients with involved nodes on pre-operative axillary ultrasound, which would fit low axillary burden criteria. The cumulative probabilities revealed that 43.2% of ultrasound positive patients have two or fewer involved nodes (17). In our study, there were 43 patients with iN+ disease, and 67.4% of them had ≤3 positive nodes. At the same time, the NCCN guideline also recommends that SLNB were applicable for patients with iN+ disease if they meet all the ACOSOG Z0011 trial criteria listed as well as low tumor burden (image-detected disease is not apparent on clinical exam and appears to be limited to one or two axillary nodes). Therefore, patients with iN+ disease might also have chance to receive dual de-escalation, including axillary surgery and systemic treatment de-escalation.

Historically, ALND used to be the standard management of axillary for SLN-positive patients, it could fully assess the overall ALNs metastases status in patients with SLN-positive disease and increase the local-regional control. Strikingly, no difference in axillary RR and OS were detected with or without ALND for early breast cancer patients with limited SLN involvement (1-2 positive SLNs) among several randomized, controlled trials such as ACSOG Z0011, AMAROS, and OTOASOR (3–5). In summary, these trials have shown that omission of ALND, followed by radiotherapy and adjuvant systemic therapy is safe and has no difference in RR in patients limited SLNs involvement (18). In the era of SLNB, axilla radiotherapy replace ALND would become the standard axillary management for patients with 1-2 positive SLNs. However, this change in the concept of axilla management prevents us from fully assessing the overall ALNs metastases status (10).

As the iconic study of 70-gene signature test, the MINDACT trial sought to provide prospective evidence that the 70-gene signature test can be used for standardizing clinical-pathological criteria in selecting breast cancer patients with up to three positive lymph node disease for avoiding adjuvant chemotherapy (19, 20). Among patients at high clinical/low genomic risk, those receiving chemotherapy had a survival without distant metastasis (DMFS) of 92.0% (95% CI: 89.6-93.8%), while those receiving no chemotherapy had a DMFS of 89.4% (95% CI: 86.8-91.5%) (21). Meanwhile, other genomic tests, such as Oncotype Dx and EndoPredict, are being recommended for analyzing chemotherapy benefit among patients with 1-3 positive nodes (22).

In the MINDACT trial, patients with negative lymph nodes underwent SLNB to obtain lymph node metastasis information, while further ALNs metastasis information for those with 1-3 positive ALNs could be obtained through ALND (23). Complete understanding of the overall ALNs metastasis status is a prerequisite for application of the 70-gene signature test among patients with positive lymph nodes. In the era of SLNB, as the omission of ALND has been widely applied for patients with 1-2 positive SLNs, we could not assess the overall ALNs metastases status of these patients through ALND. However, more than 80% of patients with 1-2 positive SLNs could also be subjected to genomic tests, and those with low genomic risk also might have chance to safely avoid chemotherapy. At the same time, according to the inclusion criteria of RxPONDER trial, patients with positive sentinel nodes were not required to undergo full ALND. The first results from RxPONDER trial revealed that the axillary surgery might not affect the iDFS of the enrolled patients (*p*-values were 0.69 and 0.26 in premenopausal and postmenopausal group, respectively) (7). It is suggested that Oncotype DX might be applied to patients with 1-2 positive SLNs without ALND. In the RxPONDER trial design, 37.4% of patients had 1-2 positive SLNs and receive no ALND. According to previous studies, nearly 20% of patients with 1-2 positive SLNs had > 3 positive ALNs. So, the probability of > 3 metastases ALNs was about 7% (37.4%×20%=7%) in the whole population of RxPONDER trial. However, as the probability was too low, the statistical power was not sufficient to detect the effective differences. With the extension of the follow-up, there might also be no difference in iDFS between different axillary surgery groups. In the era of SLNB and precision medicine, given the incomplete understanding of the overall ALN metastasis status, formulation of systemic treatment strategies requires participation of multiple disciplines including surgery, medical oncology, pathology, imaging, radiotherapy, as well as genomic risk (10). The 2021 St. Gallen consensus reported why should we accurate assessed the nodal status of HR+ patients. In the era of limited nodal information, adjuvant decision making need to know both the presence of lymph node involvement and the number of positive nodes (24). So, accurate assessment of ALNs tumor burden has important significance for optimizing the selection of suitable populations for genomic risk. The combined application of genomic risk and ACSOG Z0011/AMAROS criteria could provide patients with a better strategy of dual de-escalation treatment, which includes the de-escalation of both axillary surgery and systemic treatment.

Although the RxPONDER trial enrolled some patients with 1-2 positive SLNs without ALND, the proportion was only 37.4%. And there was no distinctive research that just enrolled patients with 1-2 positive SLNs without ALND for genomic risk. So, for these patients, there were two strategies when they want to safely apply genomic tests: one was to individually enroll patients with 1-2 positive SLNs without ALND for genomic tests, however this strategy needs to be assessed by at least 5 years of follow-up; another way was to apply our predictive nomogram to select precisely suitable populations to apply genomic tests.

This study had certain limitations. First, this retrospective database-based analysis may increase selection bias in the assignment of treatments. Second, the nomogram lacked the validation in an external population. Therefore, further prospective multi-center studies are required to confirm and assess the results of this study.

In conclusion, we created the nomogram that could be used to estimate the likelihood of having ≤3 positive ALNs based on commonly available information. The nomogram showed a good accuracy and could assist the oncologist in determining precisely which HR+/HER2- candidates with 1-2 positive SLNs without ALND could perform genomic tests. In the era of SLNB and precision medicine, the combined application of genomic tests and SLNB could provide patients with a better strategy of dual de-escalation management, including the de-escalation of both surgery and systemic treatment.

## Data Availability Statement

The raw data supporting the conclusions of this article will be made available by the authors, without undue reservation.

## Ethics Statement

Written informed consent was obtained from all patients before participation in the study. The study protocol was approved by independent ethics committees at every participating center, and the study was undertaken in full accordance with the Declaration of Helsinki.

## Author Contributions

ZB, J-JC, and P-CL were responsible for study conception and drafted the manuscript. PC and W-LW was responsible for acquisition of data and analysis. BZ was responsible for drafting of the manuscript and performed the statistical analysis. QL, JW and Y-SW conceived of the study, participated in its design and coordination, and helped to draft the manuscript. Y-BL, C-JW and P-FQ revised the manuscript. All authors contributed to the article and approved the submitted version.

## Funding

This work was funded by National Natural Science Foundation of China (81672638, 81672104), Shandong Provincial Key Research and Development Program (2017CXGC1207, 2019GSF108179, 2019GSF108104) and Shandong Cancer Hospital and Institute Clinical Training Program (20206108).

## Conflict of Interest

The authors declare that the research was conducted in the absence of any commercial or financial relationships that could be construed as a potential conflict of interest.

## Publisher’s Note

All claims expressed in this article are solely those of the authors and do not necessarily represent those of their affiliated organizations, or those of the publisher, the editors and the reviewers. Any product that may be evaluated in this article, or claim that may be made by its manufacturer, is not guaranteed or endorsed by the publisher.

## Abbreviations

HR+/HER2-, Hormone receptor positive/HER-2 negative; ALNs, Axillary lymph nodes; SLNs, Sentinel lymph nodes; ALND, axillary lymph node dissection; SLNB, Sentinel lymph node biopsy; RR, Regional recurrence; OS, Overall survival; RS, Recurrence score; iDFS, invasive disease-free survival; cN0, Clinically node-negative; iN+, Image positive node; LVI, Lympho-vascular invasion; ROC, Receiver operating characteristic; AUC, Area under the curve.
